# Executive Function Is Related to the Urinary Urgency in Non-demented Patients With Parkinson’s Disease

**DOI:** 10.3389/fnagi.2020.00055

**Published:** 2020-03-06

**Authors:** Zuzanna Tkaczynska, Sara Becker, Walter Maetzler, Maarten Timmers, Luc Van Nueten, Patricia Sulzer, Giacomo Salvadore, Eva Schäffer, Kathrin Brockmann, Johannes Streffer, Daniela Berg, Inga Liepelt-Scarfone

**Affiliations:** ^1^Department of Neurodegenerative Diseases, Hertie Institute for Clinical Brain Research, University of Tübingen, Tübingen, Germany; ^2^German Center for Neurodegenerative Diseases (DZNE), University of Tübingen, Tübingen, Germany; ^3^Department of Neurology, Christian-Albrechts-University, Kiel, Germany; ^4^Janssen Research and Development, Janssen—Pharmaceutical Companies of Johnson & Johnson, Beerse, Belgium; ^5^Reference Center for Biological Markers of Dementia (BIODEM), Institute Born-Bunge, University of Antwerp, Antwerp, Belgium; ^6^Janssen Research and Development LLC, Janssen—Pharmaceutical Companies of Johnson & Johnson, Titusville, NJ, United States

**Keywords:** Parkinson’s disease, bladder dysfunction, urge incontinence, cognition, dementia

## Abstract

**Introduction**: Evidence suggests urinary urgency is associated with cognitive impairment in a subtype of Parkinson’s disease (PD) patients. This study investigates if cognitive impairment independently predicts the presence of urinary dysfunction.

**Methods**: We report data of 189 idiopathic PD patients, excluding those with concomitant diseases or medication interacting with bladder function. A standardized questionnaire was used to define the presence of urinary urgency. All patients underwent a comprehensive motor, cognitive non-motor and health-related quality of life (HRQoL) assessment. Multivariable linear regression analysis was performed to identify independent variables characterizing urinary urgency in PD (PD-UU), which were assigned as discriminant features to estimate their individual contribution to the phenotype of the PD-UU group.

**Results**: Of 189 PD patients, 115 (60.8%) reported PD-UU. The linear regression analysis showed that among cognitive domains, executive function (EF; *p* = 0.04) had a significant negative association with PD-UU. In a second model, scores of the Montreal Cognitive Assessment (MoCA) significantly differentiated between study groups (*p* = 0.007) and also non-motor symptom (NMS) burden (*p* < 0.001). The third model consisted of reports of HRQoL, of which stigma was the only subscale of the Parkinson’s Disease Questionnaire (PDQ-39) differentiating between patients with and without PD-UU (*p* = 0.02). The linear discriminant analysis provided evidence that the combination of EF, NMS burden, nocturia, and stigma discriminated between groups with 72.4% accuracy.

**Conclusion**: In our large, non-demented PD cohort, urinary urgency was associated with executive dysfunction (EF), supporting a possible causative link between both symptoms. A combination of neuropsychological and non-motor aspects identified patients with PD-UU with high discriminative accuracy.

## Introduction

Urinary urgency is a common non-motor symptom (NMS) in Parkinson’s disease (PD; Campos-Sousa et al., 2003; Winge and Fowler, 2006; McDonald et al., 2017). Presence of urinary urgency in PD (PD-UU) lowers patients’ health-related quality of life (HRQoL; Liu et al., 2015; Moriarty et al., 2016) and its frequency is higher as among older healthy individuals (Khoo et al., 2013; Serra et al., 2018). To date, no effective treatment of PD-UU exists. The influence of dopaminergic medication is not predictable (Uchiyama et al., 2003) and standard anticholinergic medication should be avoided, for its negative impact on cognition (Ehrt et al., 2010). Therefore, novel treatment approaches are necessary.

Bladder dysfunction has been linked to a variety of clinical PD-related symptoms, including cognition (Sakushima et al., 2016; Tkaczynska et al., 2017). On the contrary, a recent study found no association between PD-UU and cognitive impairment (Picillo et al., 2017) where the applied cognitive screening assessment did not provide insight into the domain-specific characterization of cognitive functions. In vascular dementia, as well as in healthy elderly adults, the loss of executive function (EF) has been reported to be related to urinary incontinence (Haruta et al., 2013; Schumpf et al., 2017). The impairment in EF is a common feature in PD (Dirnberger and Jahanshahi, 2013), which raises the question if these specific functions associates with PD-UU in a subtype of PD patients, independent from the presence of dementia.

Our primary aim was to verify the role of domain-specific cognitive impairment, independent from the effect of other symptoms, in the occurrence of PD-UU in a large, non-demented PD sample. We hypothesized that especially EF would be associated with the presence of PD-UU. Taking limitations of previous studies into account, we excluded patients with intake of medication or the presence of concomitant diseases interacting with bladder function.

## Materials and Methods

### Patients

As a part of the ongoing “Amyloid-beta in CSF as risk factor for cognitive dysfunction in PD (ABC-PD)” study, 262 PD patients diagnosed according to the United Kingdom Brain Bank criteria (Hughes et al., 1992) were recruited. Inclusion criteria of the ABC-PD study were age between 50 and 85 years, adequate or corrected hearing/visual abilities, German as mother tongue, no history of substance abuse except for nicotine, and no further neurological diseases affecting the central nervous system. As a premise for the ABC-PD study, informed consent for a lumbar puncture was mandatory. All patients were examined during the “on” state after taking their regular optimized dopaminergic treatment.

**Figure 1:**
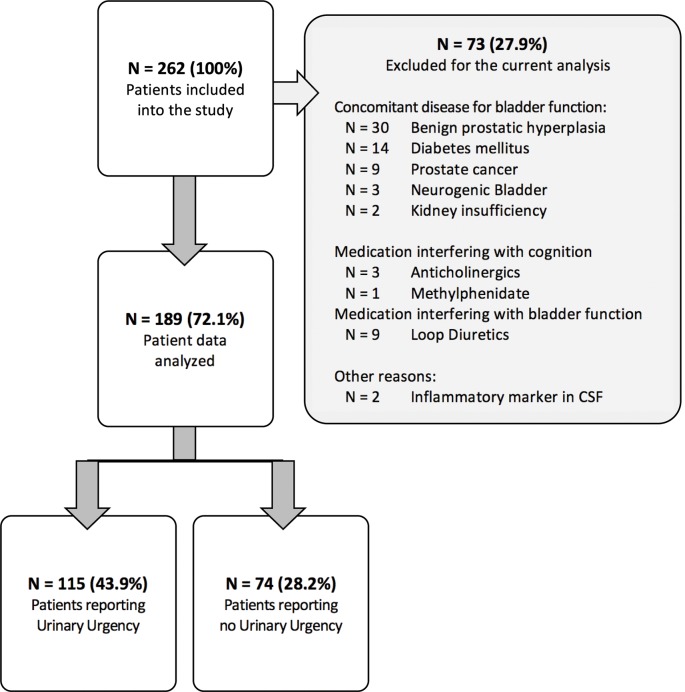
Recruitment flowchart of study groups.

For the data analysis reported here, 73 (27.9%) PD patients were excluded due to the presence of concomitant diseases affecting bladder control (see Figure 1 for details). Therefore, data of 189 PD patients were included in the final analysis. The study was approved by the local ethics committee. All patients gave written informed consent for study participation.

### Classification of PD-UU vs. PD-NUU

Item 8 “A sense of urgency to pass urine makes you rush to the toilet.” of the validated Parkinson’s Disease Non-motor Symptoms Questionnaire (NMSQuest; Storch et al., 2010) was used to differentiate between PD patients with (PD-UU, score = 1) and without (PD-NUU, score = 0) urinary urgency.

### Demographics, Medication, and Motor Symptoms

Demographics and a full drug history, including the total daily dose of all dopaminomimetics [expressed by the levodopa equivalent daily dose (LEDD; Tomlinson et al., 2010)] and the total daily dose of dopaminergic agonists medication (DAEDD) were obtained. Since medications with anticholinergic effects have been identified as a risk factor for cognitive decline, the anticholinergic risk score (ARS; Crispo et al., 2016) for medication with additional anticholinergic properties was calculated. Severity of motor symptoms was assessed by the Unified Parkinson’s Disease Rating Scale (UPDRS) part III and the Hoehn & Yahr (H&Y) scale (Goetz, 2010), presence and severity of levodopa associated motor fluctuations were assessed by item 4.3 of UPDRS part IV, while falls were assessed with item 13 of the UPDRS part II.

### Neuropsychological Test Battery

To assess major areas of cognitive function, a standardized neuropsychological test battery was applied. The Montreal Cognitive Assessment (MoCA; Nasreddine et al., 2005) was used to screen for patients’ global cognitive status. The Consortium to Establish a Registry of Alzheimer’s Disease Plus (CERAD-PLUS; Morris et al., 1989) test battery and subtests of the Wechsler Intelligence Test for adults (WIE; Von Aster et al., 2006) as well as the Performance Evaluation for Seniors (Leistungsprüfsystem, LPS-50+; Sturm et al., 1993) were assigned to the following cognitive domains: EF (CERAD-PLUS: lexical and phonemic fluency, Trail Making Test B); Attention/working memory (WIE: Digit Symbol Test, Letter Number Sequencing Task); Memory (CERAD-PLUS: Word List Learning, Word List Recall, Word List Recognition, Praxis Recall); Visuo-constructive abilities (CERAD-PLUS: Praxis, LPS-50+: Fragmented Words) and Language (CERAD-PLUS: Boston Naming Test, WIE: Similarities).

For all subtests, z-scores were computed, corrected for age (all), gender (CERAD-PLUS), and education (CERAD-PLUS) where possible. For each of the above-mentioned cognitive domains, a mean z-score was calculated. PD-related mild cognitive impairment (PD-MCI) was diagnosed based on recommendations by the Level II criteria of the Movement Disorder Society Task Force (Litvan et al., 2012).

### Further Non-motor Function Scales

Depressive symptoms were assessed with the Beck Depression Inventory-II (BDI-II; Hautzinger et al., 1995). Total HRQoL was assessed using the Parkinson’s Disease Questionnaire total score (PDQ-39; Jenkinson et al., 1997). The total score was built out of 39 questions, divided into eight subscales: Mobility (10 items); Activities of daily living (ADL, six items); Emotional well-being (six items); Stigma (four items); Social support (three items); Cognitions (four items); Communication (three items); and Bodily discomfort (three items). Other NMSs were evaluated by the German version of the PD Non-Motor Symptom Questionnaire (NMSQuest; Storch et al., 2010). The following items of the NMSQuest assessing occurrence of various NMSs were also analyzed: Item 9 “Getting up regularly at night to pass urine” to screen for nocturia; Item 19 “Finding it difficult to have sex when you try” to assess alteration of sexual behavior, and Item 23 “Difficulty getting to sleep at night or staying asleep at night” to record sleeping problems. Instrumental ADL (iADL) was measured by the Functional Activities Questionnaire (FAQ; Pfeffer et al., 1982).

### Statistics

Data were collected and managed using REDCap electronic data capture tools (Harris et al., 2009) and analyzed using SPSS 22 for Windows (SPSS Inc., Chicago, IL, USA). Descriptive values are presented as mean, standard deviation (SD), number, and percentage (%). Comparisons between groups were performed using chi-square and student’s *t*-tests, as appropriate (Table 1). To analyze the possible predictors of PD-UU, a linear regression analysis with group status (PD-UU vs. PD-NUU) as the dependent variable was applied, controlling for potential confounders (disease duration, LEDD and NMSQuest Item 9 “Nocturia”). To test the link between motor, non-motor, and cognitive symptoms and PD-UU, three independent regression models were conducted. *Model A* consisted of all five cognitive domain scores in addition to the covariates. In *model B*, the PDQ-39 summary index, NMSQuest total score, FAQ, MoCA total score, and falls incidence (UPDRS-II) were considered to evaluate the association between cognition and PD-UU among other symptoms. Given the covariance between the total score and the subscales scores of the PDQ-39, we created *model C*, in which all subscales were included as independent variables. Based on significant results from the linear regression models, a blockwise discriminant function (DF) was performed to confirm if the identified variables contributed to the classification of PD-UU. Wilks’ lambda was used to test how well each independent variable contributes to the model fit of PD-UU, and the Box-M test evaluated variance homogeneity between both groups. To validate the results of the blockwise model, a stepwise DF was calculated. The results are presented as mean and 95% confidence intervals.

**Table 1:** Demographics and clinical features of study patients.

	Total PD	PD-NUU	PD-UU	*p*-value
Number of subjects, *n* (%)	189 (100)	74 (39.2)	115 (60.8)
Male gender, *n* (%)	93 (49.2)	39 (52.7)	54 (49.5)	0.29
Age, years	64.7 (7.9)	63.5 (8.3)	65.5 (7.7)	0.09
Education, years	13.4 (2.9)	13.5 (2.9)	13.3 (2.8)	0.63
Disease duration, years	5.1 (3.8)	4.3 (3.6)	5.6 (3.9)	**0.02**
LEDD	506.4 (288.5)	441.1 (273.6)	548.9 (291.7)	**0.01**
DAEDD	166.9 (152.8)	154.6 (152.2)	174.8 (153.0)	0.37
PD-MCI n (%)	77 (40.7)	35 (47.3)	42 (36.5)	0.21
Motor performance				
UPDRS III	24.5 (10.9)	24.5 (12.7)	24.6 (9.8)	0.95
UPDRS IV				
Wearing-off, *n* (%)				0.86
0	162 (85.7)	64 (86.5)	98 (85.2)	
1	20 (10.6)	8 (10.8)	12 (10.4)	
2	6 (3.2)	2 (2.7)	4 (3.5)	
3	0 (0.0)	0 (0.9)	0 (0.0)	
4	1 (0.5)	0 (0.0)	1 (0.5)	
Hoehn & Yahr, *n* (%)				**0.06**
1	28 (14.8)	17 (22.9)	11 (9.5)	
2	110 (58.5)	38 (51.4)	72 (62.6)	
3	50 (26.4)	19 (25.7)	31 (26.9)	
4	1 (0.3)	0 (0.0)	1 (1.0)	
ARS, *n* (%)				0.35
0	66 (34.9)	31 (41.9)	35 (30.4)	
1	54 (28.6)	21 (28.4)	33 (28.8)	
2–3	45 (23.8)	14 (18.9)	31 (26.9)	
4+	24 (12.7)	8 (10.8)	16 (13.9)	
BDI-II	8.8 (7.22)	7.7 (6.63)	9.43 (7.53)	0.13
Non-motor performance				
Alteration of sexual behavior *n* (%)	59 (31.2)	16 (21.9)	43 (37.4)	**0.06**
Sleeping problems *n* (%)	92 (48.7)	29 (39.7)	63 (54.8)	0.10
Nocturia *n* (%)	119 (62.9)	37 (50.7)	82 (71.3)	0.01

*If not other indicated, values are given as mean and standard deviation. ARS, Anticholinergic Risk Scale; BDI-II, Beck Depression Inventory II; LEDD, Levodopa equivalent daily dose; DAEDD, Dopaminergic antagonist equivalent daily dose; UPDRS, Unified Parkinson Disease Rating Scale. Bold values emphasize significant p-values*.

## Results

An overview of the demographic and clinical characteristics of all 189 PD patients (93 males, 49.2%) is reported in Table 1. The mean age of all PD patients was 64.7 ± 7.9 years, with 115 patients (60.8%) reporting PD-UU. Patients reporting PD-UU had longer disease durations (*p* = 0.02), reported more frequently to experience nocturia (*p* = 0.01) and had higher LEDDs (*p* = 0.01) compared to PD-NUU. The DAEDD (*p* = 0.37) and ARS (*p* = 0.35) scores as well as patients reports of sleeping problems (*p* = 0.10) and alteration of sexual behavior (*p* = 0.06) did not significantly differ between study groups. Moreover, no group effect was found according to the presence and severity of levodopa associated motor fluctuations (*p* = 0.86). The frequency of patients with a diagnosis of PD-MCI did not statistically differ between groups.

Regarding the linear regression (results are placed in Table 2), among all cognitive domains (*model A*), PD-UU patients performed worse on tests assessing EF (*p* = 0.04), but not on other domains compared to PD-NUU. In *model B*, the MoCA score (*p* = 0.007) and the total score of NMSQuest (*p* < 0.001) significantly differentiated between study groups, whereas the FAQ, PDQ-39 total score, and falls rate did not statistically contribute to the prediction of PD-UU. Values of the Stigma subscale of the PDQ-39 (*model C*) were lower among PD-UU patients compared to PD-NUU (*p* = 0.02) and were considered as the only subscale associated with the presence of urinary urgency. The cofounders did not reach the significance level in any of the regression models.

**Table 2:** Cognitive performance and non-motor features of study patients.

Domains	Total PD	PD-NUU	PD-UU	Standardized beta	95% CI	*p*-value
Model A*				
Executive function	−0.16 (0.92)	−0.03 (0.93)	−0.43 (0.90)	0.51	(0.84–2.16)	**0.04**
Attention	−0.11 (0.76)	−0.18 (0.75)	−0.11 (0.76)	-0.19	(0.47–1.45)	0.41
Language	−0.21 (0.78)	−0.18 (0.74)	−0.27 (0.79)	0.19	(0.87–2.23)	0.25
Memory	−0.25 (0.87)	−0.39 (0.76)	−0.16 (0.92)	0.34	(0.07–1.89)	0.68
Visuo-constructive skills	−0.34 (0.86)	−0.43 (0.88)	−0.28 (0.79)	0.04	(0.67–1.61)	0.73
Model B*				
PDQ-39 total score	4.2 (3.6)	3.8 (3.3)	4.5 (3.7)	-0.13	(0.76–1.02)	0.14
FAQ	2.1 (2.1)	2.0 (2.9)	2.2 (4.1)	-0.02	(0.86–1.12)	0.78
MOCA	26.30 (3.34)	26.11 (3.18)	24.14 (3.18)	0.15	(1.04–1.30)	**0.007**
NMSQuest	7.7 (4.7)	5.4 (4.3)	9.2 (4.4)	0.29	(1.19–1.51)	**0.001**
UPDRS II: falls	0.25 (0.62)	0.16 (0.46)	0.34 (0.66)	0.28	(0.57–3.24)	0.52
Model C*				
PDQ-39 mobility	17.2 (19.3)	13.4 (17.3)	19.6 (20.2)	0.002	(0.98–1.02)	0.89
PDQ-39 ADL	20.7 (18.3)	16.7 (16.5)	23.5 (19.2)	0.02	(0.99–1.05)	0.09
PDQ-39 emotional well-being	17.2 (17.9)	14.6 (19.5)	18.7 (18.8)	0.010	(0.98–1.04)	0.48
PDQ-39 stigma	14.7 (18.5)	16.3 (20.6)	13.6 (17.1)	-0.029	(0.94–99)	**0.02**
PDQ-39 social support	10.7 (18.5)	9.6 (17.2)	11.4 (19.3)	-0.001	(0.98–1.02)	0.96
PDQ-39 cognitions	10.7 (18.4)	15.2 (15.3)	29.5 (17.9)	-0.005	(0.98–1.04)	0.64
PDQ-39 communication	17.6 (19.3)	15.8 (18.9)	18.8 (19.5)	0.013	(0.97–1.02)	0.70
PDQ-39 bodily discomfort	23.7 (21.8)	19.5 (20.2)	26.3 (22.5)	-0.758	(0.99–1.03)	0.18

All values are given as z-values: median and standard deviation; ADL, Activity of Daily Living Function; FAQ, Functional Activities Questionnaire; MOCA, Montreal Cognitive Assessment Test; NMSQuest, Non-motor Symptoms Questionnaire; PDQ-39, Parkinson’s Disease Questionnaire; UPDRS II, Unified Parkinson Disease Rating Scale II; *Every model also consisted of cofounders, which are not listed in the table. Bold values emphasize significant p-values.

To predict group membership of PD-UU, we executed a discriminant analysis. Predictor variables were the z-score of the EF domain, MoCA total score, NMSQuest total score, and the PDQ39-Stigma scale score. Based on the blockwise model, PD-UU patients were classified with an accuracy of 72.4% for group discrimination. Box’s M (*p* = 0.81) and Wilks’ lambda (*λ* = 0.73, *χ*^2^ = 57.9, *df* = 5, *p* < 0.001) confirmed a high quality of model fit resulting in the following DF: DF = (1.1 × NMSQuest total score) + (0.47 × EF z-score) + (0.1 × NMSQuest Item 9 “Nocturia”) − (0.63 × PDQ-39 subscale score “Stigma”); (*p* < 0.001). Due to its low discriminant power, the MoCA score was not significant in the DF. In the discriminant model, the pooled within-groups correlations between discriminating variables and standard canonical DF built the following hierarchy: NMSQuest total score differentiated best between PD-UU and PD-NUU (*p* < 0.001), followed by EF z-score (*p* = 0.02), then by the NMSQuest Item score “Nocturia” (*p* = 0.02) and PDQ-39 subscale score “Stigma” (*p* = 0.02). The stepwise analysis confirmed that all variables together, apart from the MoCA, contributed significantly (*p* < 0.001) to group discrimination of PD-UU and PD-NUU.

## Discussion

We here present results of a study investigating the link between urinary urgency and cognitive impairment in a selected cohort of PD patients controlled for the intake of concomitant medication and the presence of age-related bladder symptoms. Our most important finding is the prediction of urinary urgency with high accuracy, given the combination of NMS burden, executive dysfunction, nocturia and self-perceived stigma in PD.

During the course of the disease, presence of NMS start to predominate the clinical picture, which is often more troublesome for patients than the motor symptoms (Martinez-Martin et al., 2011). Hence, it is important to detect the causal relation of the non-motor burden to develop more specific adjuvant therapy forms.

Our data shows that cognitive impairment, especially EF, is associated with the occurrence of PD-UU among other motor and non-motor symptoms. Some reports suggest an interdependence between autonomic and cognitive symptoms, which was shown for orthostatic hypotension and cognitive worsening (Heims et al., 2006). However, the association between PD-UU and cognition is only sparsely investigated despite a high prevalence rate of urinary urgency in some forms of dementia or late stages of PD (Del-Ser et al., 1996; McKeith et al., 2005; Ransmayr et al., 2008; Tateno et al., 2015). PD-UU can be caused by the neurodegenerative processes of the prefrontal cortex since the frontal cortex-basal ganglia circuit plays a prominent role not only in modulating EF and goal-directed behavior (Funahashi, 2001) but also in suppressing micturition (Winge, 2015). Neuroimaging studies identified that the prefrontal cortex is activated during bladder filling (Kitta et al., 2016) and that progressive neurodegenerative changes in PD cause disruptions of these patterns (Kitta et al., 2006; Fowler and Griffiths, 2010). The causality of connection between EF and PD-UU should be considered as bidirectional, as executive impairment might additionally prevent PD patients from planning or inhibiting physiological processes that lead to PD-UU.

The association between PD-UU and cognition as a consequence of cerebrovascular burden cannot be excluded, given the age of subjects and their disease duration. Vascular Parkinsonism accounts for 4–12% of all cases, however, comorbidity can be observed in up to 44% (Jellinger, 2003; Thanvi et al., 2005; Turnbull, 2005; Buchman et al., 2011). Moreover, cerebrovascular lesions contribute to cognitive worsening and increase the risk of dementia (Burton et al., 2006; Boomsma et al., 2019). The studies including sensitive imaging techniques are needed to specify this connection.

In this study, PD-UU was reported by 60.8% of non-demented PD patients. This finding demonstrates that PD-UU is not limited to a subgroup of demented PD patients or PD-MCI, but can develop early and correlate with early cognitive deteriorations. The rate of patients with and without PD-MCI did not differ in our sample, which emphasizes that cognitive impairment *per se* does not lead to the onset of PD-UU. Rather, our data suggest that a specific cognitive function is associated with the presence of PD-UU, at least in a subtype of PD patients.

In our study, we used a comprehensive neuropsychological battery, including tests for the assessment of cognitive domains. This approach might have allowed us to detect prefrontal cognitive changes that other studies could not observe (Picillo et al., 2017). Performance in the MoCA did distinguish between our study groups, however, it did not independently contribute to the classification of PD-UU in a DF analysis. The MoCA is a global cognitive measure with a low sensitivity for the assessments of deficits in single cognitive domains. The substantial overlap with the assessed EF tests might have weakened its discriminative role of the MoCA in further analysis. Contrary to our findings, in the PRIAMO cohort, no association between urinary dysfunction and the global cognitive assessment, measured by Mini-Mental State Examination (MMSE), independently from the presence and severity of other non-motor symptoms, could be identified (Picillo et al., 2017). Further studies are necessary to specify the distinctive mechanism of executive dysfunction potentially causing PD-UU, specifically considering the prefrontal area as a control center for decision-making or inhibitory function in PD (Sakagami et al., 2006; Haruta et al., 2013). The impairment in EF is a common and early feature in PD (Dirnberger and Jahanshahi, 2013), which raises the question if these specific functions might be associated with urinary urgency (with or without incontinence) in a subtype of PD patients. EF is only sparsely assessed within the MMSE, which might explain divergent results among previous studies evaluating the association between cognition and PD-UU.

Apart from the more pronounced executive worsening, patients with PD-UU reported higher non-motor symptom burden. This confirms previous findings reporting non-motor symptoms as highly prevalent in PD patients (Pfeiffer, 2016) and that PD-UU patients experience higher rates of non-motor symptoms than PD-NUU (Winge, 2015; Picillo et al., 2017). PD is considered a spectrum disorder, whereas non-motor symptoms have been recognized as a progression marker of the disease (Chaudhuri et al., 2015; Marras and Chaudhuri, 2016). The significantly higher prevalence of non-motor symptoms in the PD-UU subgroup in our study shows that PD-UU might influence the experienced effect on PD-UU and/or other non-motor symptoms (Picillo et al., 2017). Indeed, our findings are in line with previous studies that showed that autonomic dysfunction should be considered a risk factor for a more progressive course of PD (Jain, 2011; Kotagal et al., 2016). Hence, the assessment of various non-motor symptoms is needed to subtype the heterogeneity of PD patients.

Regarding the iADL function, no difference defining the status of PD-UU was found. Previous studies have shown that the presence and severity of urinary symptoms might have the potential to alter patients’ everyday behavior, mainly due to the withdrawal of their social activities (Moriarty et al., 2016). Based on the assessment of the FAQ and the ADL subscale of the PDQ-39, we were not able to support these previous results (Liu et al., 2015). The HRQoL related to stigma differed between PD-UU and PD-NUU but was contrary to our expectations. The reverse direction of the stigma outcome may imply that PD-UU patients have difficulties perceiving the full consequences of urinary urgency or may tend to deny these symptoms, as PD patients suffer from a reduced interoceptive sensitivity, compared to a healthy population (Ricciardi et al., 2016). The interoceptive sensations seem to be modulated by the anterior cingulate cortex, a region that has been also associated with the performance in executive tasks (Paus et al., 1998; Fowler and Griffiths, 2010). To gain further insight into this connection, more studies are required.

In line with previous findings using either questionnaires or urodynamic tests, our PD population presented a high prevalence of PD-UU (Araki et al., 2000; McDonald et al., 2017; Picillo et al., 2017). No differences were observed in our study groups regarding sleep problems or fluctuation of levodopa response, which could influence the subjective perception of a discomfort related to urinary tract symptoms (Campos-Sousa et al., 2003; Uchiyama et al., 2003, 2011; Breen and Drutyte, 2013; Smith et al., 2015). Sexual symptoms were reported in 31% of all patients (Sakakibara et al., 2011), but the distribution did not differ among our study groups. It leads us to the conclusion, that sexual symptoms are not a variable characterizing PD-UU patients.

Adjusting for factors relating to comorbidities that may play a role in developing age-related bladder symptoms, we minimized the possible misclassification and concentrated on urinary urgency as an independent medical condition caused by PD. However, we cannot rule out that some patients, who are primarily diagnosed with idiopathic PD, will develop atypical PD, within the disease course known for vaster neurodegenerative processes (Hughes et al., 2002). As prevalence rate is comparable to other studies using a self-rated questionnaire (Winge et al., 2006; Winge, 2015), and the median disease duration was around 5 years, we do think that this is true for the majority of our patients.

In this study presence of PD-UU was associated with longer disease duration, a higher dosage of LEDD, which has been previously reported (Liu et al., 2015; Winge, 2015), but neither to age nor gender. We found nocturia to be reported as in previous studies- about 60% prevalence rate (Yeo et al., 2012; Smith et al., 2015; Winge, 2015). The PD-UU patients reported nocturia significantly more often than PD-NUU patients, showing that both, nocturia and urgency are associated with disease duration (Araki et al., 2000). The number of urinary symptoms was shown to be rising with disease duration (Winge and Nielsen, 2012) and even though, PD-UU patients had higher LEDD, they reported higher nocturia prevalence, which shows that unlike motor symptoms urinary dysfunctions are more difficult to manage with levodopa, suggesting a complex pathomechanism (Sakakibara et al., 2010; Uchiyama et al., 2011). This issue should be addressed in future studies in order to raise patients’ HRQoL.

To reduce the possible statistical bias, we corrected for potential confounders in all steps of our analysis. Even though disease duration and LEDD differentiated between our study groups, they did not contribute as independent traits of PD-UU, as their predictor value was not significant in any of our regression models. This argues for our assumption that PD-UU is a symptom strongly dependent upon the occurrence of other non-motor symptoms, characterizing a specific PD subtype. PD-UU, when diagnosed as an intrinsic PD symptom, rather than age-related comorbidity, can be an important diagnostic clue for the disease progression and supports the existence of the autonomic PD subtype (Marras and Chaudhuri, 2016; Sauerbier et al., 2016). However, the neuropathological pathway and its correlatives are not fully explained yet.

This study has limitations. The cross-sectional study design does not allow exploration of the causative nature of the link between urinary urgency and cognition completely. A prospective longitudinal study design would help to identify a causative association and help to understand the development of these PD non-motor symptoms. Nevertheless, the applied statistical method allowed us to assess objective classification to a specific group, taking this limitation into concern. The non-demented patients were mostly recruited from a study that required a lumbar puncture, which could have biased the recruitment of the participants. In general, we were able to replicate previously reported prevalence rates of PD-UU and associations with these phenomena to more advanced disease stages (Winge and Nielsen, 2012; Winge, 2015; Picillo et al., 2017). However, even though our data replicates prevalence from previous research, we cannot exclude the possibility that we indeed had false positive and false negative subjects, as no urodynamic examination was concluded.

Secondly, PD patients were assigned to the PD-UU or PD-NUU group based on a self-reported questionnaire. This design introduces a way to identify PD-UU in a highly economical way- for the used questionnaire is easy to implement in a daily clinical routine. However, even though our data replicates prevalence from previous research (Araki et al., 2000; Sakakibara et al., 2011; Picillo et al., 2017), we cannot exclude the possibility that we indeed had false positive or false negative subjects, as no urodynamic examination was concluded. In order to minimize cofounding data, this study had strict inclusion criteria, which led to a high number of exclusions. This design was needed to investigate the urinary urgency caused by PD and its neurodegenerative deterioration. Otherwise, the presence of concomitant, age-related diseases causing bladder dysfunction could bias the results. Future studies using a semi-structured interview, or urodynamic examination are needed to improve the characterization of the association between EF and urinary symptoms in PD.

In conclusion, to our knowledge, this is the largest study that investigates the role of cognitive impairment in PD-UU using a comprehensive neuropsychological test battery. Our findings emphasize that cognition should be taken into consideration as a predictor for urinary urgency, offering an alternative opportunity for intervention strategies.

## Data Availability Statement

The datasets generated for this study are available on request to the corresponding author.

## Ethics Statement

The studies involving human participants were reviewed and approved by Ethic Comittee of University of Tübingen. The patients/participants provided their written informed consent to participate in this study.

## Author Contributions

ZT: organization and execution of research project, design and execution of data analysis and writing of the first manuscript draft. SB and ES: execution of research project, review and critique. WM, MT, LV, PS, GS, KB, JS and DB: review and critique. DB: conception of the research project, review and critique. IL-S: conception and organization of the research project, conception and supervision of statistical analysis, review and critique.

## Conflict of Interest

ZT Landesgraduiertenförderungsgesetz (LGFG) University of Tübingen. SB reports no disclosures. WM holds part of a patent for the assessment of dyskinesias (German patent office, 102015220741.2); and is an advisory board member for Access and Pricing Strategy GmbH and Abbvie. He receives honoraria from Abbvie, UCB, Rölke Pharm and grants from the European Union, the Michael J. Fox Foundation, Neuroalliance, Lundbeck and Janssen—Pharmaceutical Companies of Johnson & Johnson. MT is a fulltime employee of Janssen Research and Development, Janssen—Pharmaceutical Companies of Johnson & Johnson. Owns shares of Johnson & Johnson. LV is a fulltime employee of Janssen Research and Development, Janssen—Pharmaceutical Companies of Johnson & Johnson. PS reports no disclosures. GS is a fulltime employee of Janssen Research and Development LLC, Janssen—Pharmaceutical Companies of Johnson & Johnson, Titusville, NJ, USA. ES received a research grant from the University of Kiel, Germany (Research Rotation position). KB received a research grant from the University of Tuebingen (TUEFF) and the German Society of Parkinson’s disease (dPV), funding from the Michael J. Fox Foundation (MJFF), travel grants from the Movement Disorders Society and speaker honoraria from Lundbeck and Zambon. JS is a former full-time employee of Janssen Research and Development, Janssen—Pharmaceutical Companies of Johnson & Johnson. He is currently employed by UCB Pharma, Antwerpen, Belgium. DB has served on scientific advisory boards for Novartis, UCB/SCHWARZ PHARMA, Lundbeck, and Teva Pharmaceutical Industries Limited; has received funding for travel or speaker honoraria from Boehringer Ingelheim, Lundbeck Inc., Novartis, GlaxoSmithKline, UCB/SCHWARZ PHARMA, Merck Serono, Johnson & Johnson, and Teva Pharmaceutical Industries Limited; and has received research support from Janssen, Teva Pharmaceutical Industries Limited, Solvay Pharmaceuticals, Inc./Abbott, Boehringer, UCB, Michael J. Fox Foundation, BMBF, dPV (German Parkinson’s disease association), Neuroallianz, DZNE and Center of Integrative Neurosciences. IL-S reports grants from the International Parkinson Funds (Deutschland) GmbH (IPD), Michael J. Fox Foundation, Johnson & Johnson and European Commission, H2020-TWINN-2015. At the time of conduction of the study, data analyses, and manuscript preparation, MT, LV, GS and JS were employees of Janssen—Pharmaceutical Companies of Johnson & Johnson.
